# ELECTIVE TELEMEDICINE: ARE OLDER WOMEN IN THE POSTPRIMARY TREATMENT PHASE OF EARLY BREAST CANCER INTERESTED?

**DOI:** 10.1093/geroni/igac059.2218

**Published:** 2022-12-20

**Authors:** Caroline Buse, Kirsten Nyrop, Erin O'Hare Kelly, Hyman Muss

**Affiliations:** UNC Chapel Hill, Chapel Hill, North Carolina, United States; UNC Chapel Hill, Chapel Hill, North Carolina, United States; UNC Chapel Hill, Chapel Hill, North Carolina, United States; UNC Chapel Hill, Chapel Hill, North Carolina, United States

## Abstract

Explosive growth in the use of telemedicine occurred during the COVID-19 pandemic. Telemedicine has the potential to lessen healthcare burden for older adults with frequent appointments, physical and cognitive disabilities, and reliance on caretakers. To benefit from telemedicine, patients must have the capacity to engage with technology, for which inexperience and access may pose barriers. This study aimed to better understand the perspectives of older women with non-metastatic breast cancer on telemedicine, in regards to visit convenience, completeness, and interpersonal satisfaction. In this qualitative study, semi-structured interviews were conducted in a convenience sample of women age 65+, post-primary treatment for Stage I-III breast cancer, who received in-person outpatient care at NC Cancer Hospital before transitioning to telemedicine after March 2020. Patients were interviewed about their perceptions of telemedicine (telephone, video) as compared to in-person visits. Audio files of interviews were transcribed and reviewed to identify themes established a priori in the interview protocol. 15 patients (telephone=5, video=10) were consented and interviewed (July-October 2021), mean age=74. 13/15 participants reported that they preferred a hybrid care model that included telemedicine care over in-person care alone. COVID-19, physical disability, and transportation burden were associated with telemedicine preference. Comfort with familiar patient-provider interaction and lack of physical exam were associated with in-person appointment preference. Patient-clinician conversations and clinic protocols guiding use of telemedicine should take into account newness of diagnosis, patient comfort and familiarity with the care team, travel burden, disability, and whether the physical exam is or is not essential.

